# Preconceptional smoking alters spermatozoal miRNAs of murine fathers and affects offspring’s body weight

**DOI:** 10.1038/s41366-021-00798-2

**Published:** 2021-05-17

**Authors:** Barbara Hammer, Latha Kadalayil, Eistine Boateng, Dominik Buschmann, Faisal I. Rezwan, Martin Wolff, Sebastian Reuter, Sabine Bartel, Toril Mørkve Knudsen, Cecilie Svanes, John W. Holloway, Susanne Krauss-Etschmann

**Affiliations:** 1 Early Life Origins of Chronic Lung Disease, Research Center Borstel, Leibniz Lung Center, German Center for Lung Research (DZL), Borstel, Germany; 2 Human Development and Health, Faculty of Medicine, University of Southampton, University Hospital Southampton, Southampton, UK; 3 Division of Animal Physiology and Immunology, TUM School of Life Sciences Weihenstephan, Technical University of Munich, Freising, Germany; 4 School of Water, Energy and Environment, Cranfield University, Cranfield, Bedfordshire UK; 5 Department of Pulmonary Medicine, Experimental Pneumology, University Hospital Essen - Ruhrlandklinik, Essen, Germany; 6 University of Groningen, University Medical Center Groningen, Department of Pathology and Medical Biology, GRIAC Research Institute, Groningen, The Netherlands; 7 Department of Clinical Science, University of Bergen, Bergen, Norway; 8 Department of Occupational Medicine, Haukeland University Hospital, Bergen, Norway; 9 Centre for International Health, Department of Global Public Health and Primary Care, University of Bergen, Bergen, Norway; 10 NIHR Southampton Biomedical Research Centre, University Hospital Southampton, Southampton, UK; 11 Institute for Experimental Medicine, Christian-Albrechts-Universität zu Kiel, Kiel, Germany

**Keywords:** Cell biology, Genetics, Metabolism

## Abstract

**Background:**

Active smoking has been reported among 7% of teenagers worldwide, with ages ranging from 13 to 15 years. An epidemiological study suggested that preconceptional paternal smoking is associated with adolescent obesity in boys. We developed a murine adolescent smoking model before conception to investigate the paternal molecular causes of changes in offspring’s phenotype.

**Method:**

Male and female C57BL/6J mice were exposed to increasing doses of mainstream cigarette smoke (CS) from onset of puberty for 6 weeks and mated with room air (RA) controls.

**Results:**

Thirteen miRNAs were upregulated and 32 downregulated in the spermatozoa of CS-exposed fathers, while there were no significant differences in the count and morphological integrity of spermatozoa, as well as the proliferation of spermatogonia between CS- and RA-exposed fathers. Offspring from preconceptional CS-exposed mothers had lower body weights (*p* = 0.007). Moreover, data from offspring from CS-exposed fathers suggested a potential increase in body weight (*p* = 0.062).

**Conclusion:**

We showed that preconceptional paternal CS exposure regulates spermatozoal miRNAs, and possibly influences the body weight of F1 progeny in early life. The regulated miRNAs may modulate transmittable epigenetic changes to offspring, thus influence the development of respiratory- and metabolic-related diseases such as obesity, a mechanism that warrants further studies for elaborate explanations.

Teenage smoking is at a remarkably high rate with a prevalence of 7% among 13–15 year olds worldwide and 5–17% across Europe [1]. Paternal preconceptional environmental exposures recently emerged as new risk factors threatening the health of future offspring [2–4]. Additionally, preconceptional smoking has been described to alter spermatozoal miRNAs of murine fathers, leading to changes in offspring’s body weight. Potential vectors of intergenerational information include epigenetic changes in germ cells such as DNA methylation [5], histone/protamine modifications, and miRNA patterns [6]. Existing data relate paternal diet to epigenetic signatures and consequently, intergenerational influences on embryogenesis and metabolic changes in offspring [7, 8]. Moreover, paternal smoking onset before puberty was recently related to adolescent obesity of male offspring [9]. Furthermore, obesity during puberty has been linked to increased asthma risk in future children [10, 11]. Interestingly, a recent epidemiological study indicated that offspring of fathers who smoked, particularly before the age of 15, was potentially at risk of developing asthma [12]. These studies highlight the importance of preconceptional paternal smoking to offspring’s health, and identification of the molecular mechanism underlying these effects could provide information on potential targets for secondary prevention of disease in early life.

To investigate the potential harm of prepubertal and pubertal cigarette smoking on the risk of obesity in offspring, we exposed 3-week-old male and female mice to room air (RA) or increasing doses of cigarette smoke (CS) for 6 weeks, followed by mating with nonsmoking partners. CS-exposed mothers and nonsmoking parents served as positive and negative controls, respectively (see online repository, Supplementary Fig. 1a). Mice mimicking smoking, future fathers and mothers had significantly lower weight gain compared to RA controls of the corresponding sexes (Supplementary Fig. 1b, c). As expected, differential cell counts in bronchoalveolar lavage fluid revealed elevated levels of macrophages and neutrophils after CS exposure (Supplementary Fig. 2a, b). These observations give insights into the quality of parental CS exposure in the lungs. In addition, in a different set of experiments, increased cotinine levels were detected in the serum of male (Supplementary Fig. 2c) and female animals [13] exposed to comparable doses of CS during adulthood as to mice in the present study. Based on these observations as well as previous data [13], this corresponds approximately to moderate-to-heavy smoking. The count and morphological integrity of spermatozoa as well as the proliferation of their progenitor cells (spermatogonia) in the seminiferous tubules of testes showed no differences between RA- and CS-exposed future fathers (Supplementary Fig. 3a–d).

In this preliminary study, we hypothesized that molecular changes in the male germ line could intergenerationally influence the phenotype of offspring. To address this question, sperm cell miRNAs were analyzed by next-generation sequencing in RA- and CS-exposed fathers. This identified the differential expression of a diverse pool of 13 upregulated and 32 downregulated miRNAs associated with the regulation of several functional and structural processes, in the spermatozoa of mice following CS exposure (Fig. 1A, B). For example, miR-204-5p, miR-96-5p, and miR-340-5p, which were differentially expressed in response to CS (Supplementary Table 1 and Supplementary Fig. 4), were found to be associated with tissue morphogenesis and development. Furthermore, KEGG pathway analysis associated miR-204-5p and miR-96-5p differential expression to the Hippo and Estrogen signaling pathways, which are involved in lung and early embryo development, respectively [14, 15]. The Hippo signaling pathway also maintains the pluripotency of stem cells during early embryogenesis [16, 17], which, in turn, determines the fate of organ development. In addition, dysregulation of miR-340-5p together with three other miRNAs (miR-133b-3p, miR-196a-5p, and miR-205-5p) has been previously reported in the sperm of fathers who received a high-fat diet, and were associated with the transmission of obesity and insulin resistance predominantly through two generations of female offspring [18]. Together, our results suggest a role for the CS-induced miRNAs identified in this study, in organ development, and early embryogenesis. Further studies are warranted to unravel the posttranscriptional role of miR-340-5p in the spermatozoa of smoking mouse models.

**Fig. 1:**
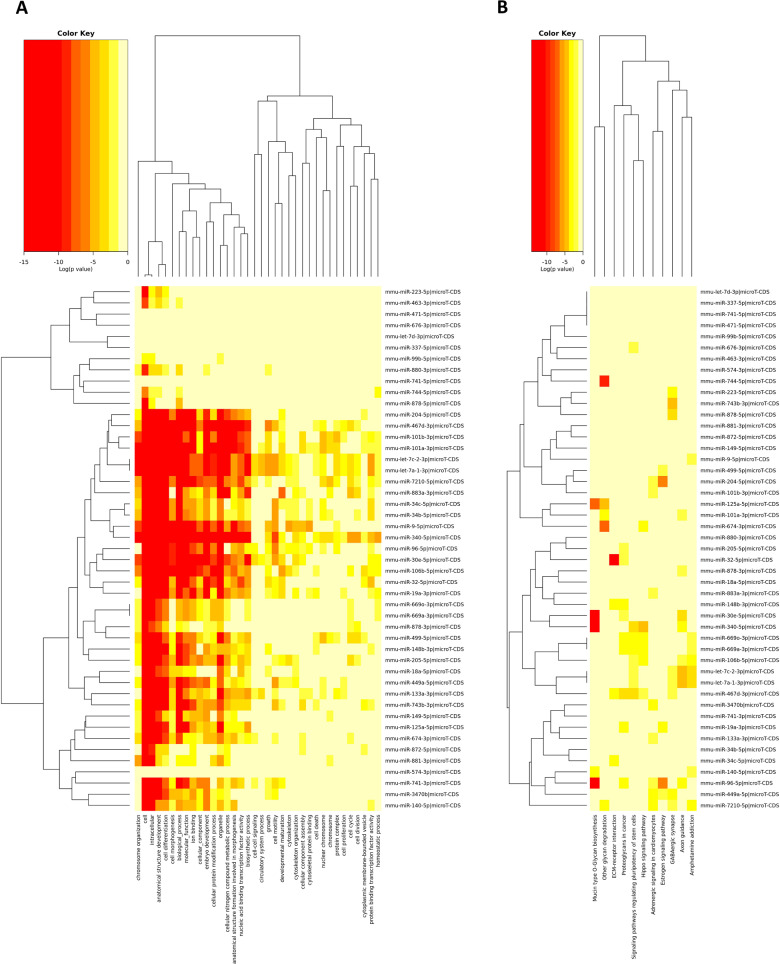
Profile of miRNAs in the spermatozoa from RA- compared to CS-exposed mice. Heatmaps representing gene ontology (**A**) and KEGG (Kyoto Encyclopedia of Genes and Genomes) pathway analyses (**B**). Data were generated from one experiment giving a total of 4 (RA) and 5 (CS) animals per group.

Collectively, offspring of CS-exposed mothers had lower body weight (mean for 21 days) than those of nonsmoking mothers (*p* value = 0.007, Table 1). Importantly, this data highlights the potential relevance of preconceptional smoking as a risk period for offspring’s health, as in human studies, it is difficult to separate preconceptional and *in utero* effects of maternal smoking. We did not observe any statistically significant difference between the offspring’s mean body weight of RA- and CS-exposed fathers, although the skewed confidence interval (CI) suggests a possible increase in body weight for the latter (95% CI: −0.01, 0.23, Table 1). Nevertheless, our findings indicate altered growth, which corroborate with existing epidemiological data, that is, obesity in relation to father’s prepuberty smoking [9]. To investigate if body weight is characterized by metabolic changes, we profiled genes coding for proteins related to metabolic functions, adipogenesis, and glucose metabolism in the liver. At postnatal day 21, the gene profiles of *Il6*, *Adipoq*, *Lepr*, *Insr*, and *Pparg* were similar in offspring from CS-exposed fathers or mothers compared to offspring of nonsmoking parents (Supplementary Fig. 5a–e).

**Table 1:** Comparison of offspring’s body weight from smoking and nonsmoking parents.

	*n*	Coefficient	95% CI	*p* value
Mean birth weight	72	1.16	0.99, 1.32	<0.001
Days	72	0.43	0.41, 0.44	<0.001
Paternal CS^a^	30	0.11	−0.01, 0.23	0.062
Maternal CS^a^	22	−0.18	−0.30, −0.05	0.007
Sex^b^	72	−0.01	−0.10 to 0.08	0.830

Results of multivariate linear mixed-effect analysis of a three-level (litter, offspring, and days) data set on offspring’s weight based on linear regression are shown. The weight of each offspring was measured daily for 21 days. The linear regression model included sex of the offspring and parental exposure (CS) as fixed factors and days as a random factor. *p* values reported for parental exposure are adjusted for sex of the offspring and *p* value for sex of the offspring is adjusted for parental CS exposure (see Supplementary Methods). Experiments were performed three times independently. The analysis included 30 pups of parents exposed to RA, 22 pups where only mothers were exposed to CS and 30 pups where only fathers were exposed to CS.

*n* number of offspring in the analyses, *days* 0–21 (0: day of birth), *CI* confidence interval, *CS* cigarette smoke, *RA* room air.

^a^Reference group: nonsmokers (*n* = 20).

^b^Reference group: females (*n* = 36).

We understand that the smoking period after completion of puberty and the nonsmoking period during mating might be a limitation to our study. Nonetheless, our data are in agreement with previously reported epidemiological findings [9]. The observed body weight differences in the progeny need to be explored in follow-up studies, with considerations for fat tissues and muscle mass analyses. To explain the regulatory roles of the selected miRNAs along with risk of metabolic alterations in offspring, microinjection of the corresponding miRNA mimics and inhibitors into the cytoplasm of early zygotes could provide valuable molecular insights. Furthermore, the mechanisms by which maternal smoking during puberty affects the weight of the offspring, although not being the subject of this study, need further investigation. In summary, this preliminary study demonstrates that preconceptional paternal CS exposure modifies the expression of miRNAs in spermatozoa and possibly influences the body weight of F1 progeny in early life. Thus, miRNAs in the plasma microenvironment of spermatozoa may represent a mechanism for transmittable epigenetic changes to offspring and development of metabolic or respiratory diseases, further highlighting paternal smoking as potential risk factor for offspring’s health.

## Supplementary information

Supplemental material
