# Supplementary material for: Preconceptional smoking alters spermatozoal miRNAs of murine fathers and affects offspring’s body weight
Source: Int J Obes (Lond). 2021 May 17;45(7):1623–7. doi: 10.1038/s41366-021-00798-2 (PMC8236406; doi:10.1038/s41366-021-00798-2)
Supplement: Supplementary file 1 — Supplemental material [file 41366_2021_798_MOESM1_ESM.pdf]

Supplementary Material

Supplementary Figures

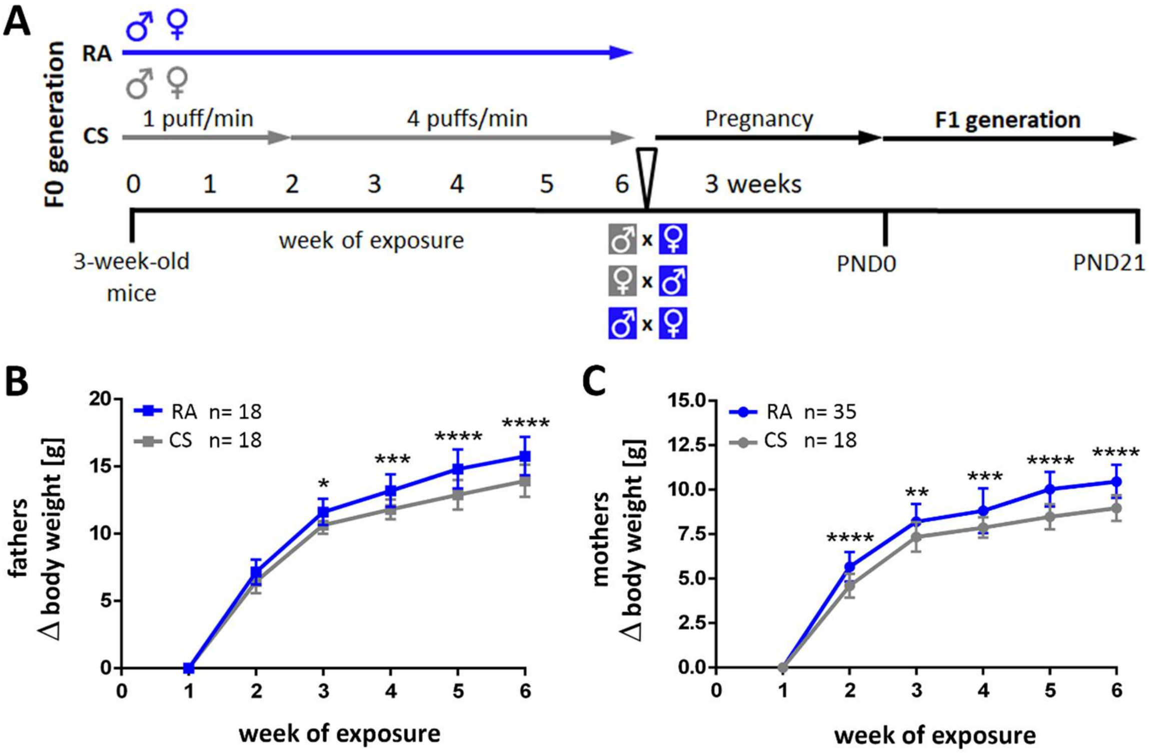

Supplementary Figure 1: Paternal and maternal preconceptional CS exposure. Schema of animal model used. Mating was performed for 48 h after RA or CS exposure (A). Body weight gain (Δ body weight) of future fathers (B) and mothers (C) during exposure to RA or CS. Two-way ANOVA with Sidak's multiple comparisons test (B-C). Data are expressed as mean ± SD. Experiments were performed three times independently, giving a total of 18 (RA and CS-exposed fathers, CS-exposed mothers) and 35 (RA-exposed mothers) animals per group. RA: room air, CS: cigarette smoke. * $p < 0.05$, ** $p < 0.01$, *** $p < 0.001$, **** $p < 0.0001$.

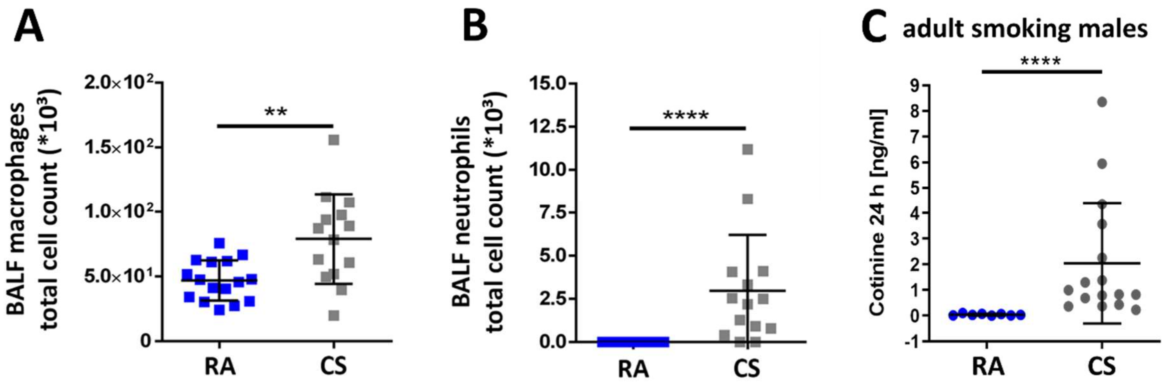

Supplementary Figure 2: Analysis of inflammatory cells in BALF of lungs from RA and CS-exposed fathers. Quantitative analyses of BALF macrophages (A) and neutrophils (B) of fathers (■ n= 16, ■ n= 14). In a different set of experiments, detection of serum cotinine in adult males exposed to RA or CS (4 puffs/min, 24 days, 1 h/day, 7 days/week) 24 h after exposure (● n= 8, ● n= 16) (C). Data are compared by Unpaired student's *t* test (A) or Mann-Whitney *U* test (B, C). Data are represented as mean ± SD. Experiments were performed three times independently, giving a total of animals per group as described above. Each data point represents an individual animal. BALF: Bronchoalveolar lavage fluid, RA: room air, CS: cigarette smoke. * $p < 0.05$, ** $p < 0.01$, *** $p < 0.001$, **** $p < 0.0001$.

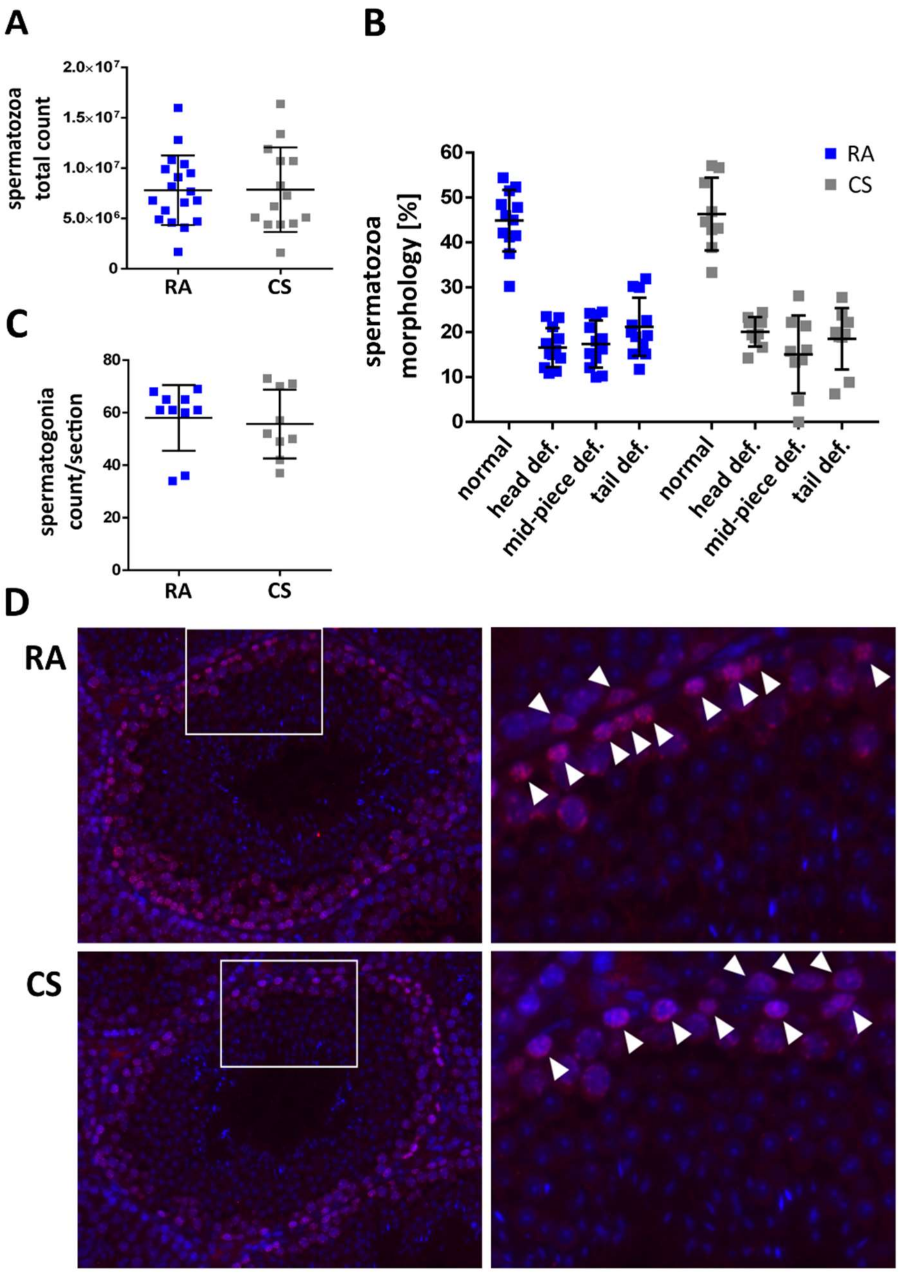

Supplementary Figure 3: Analysis of spermatozoa and spermatogonia in murine fathers. Quantitative analyses of sperm count (■ n= 18, ■ n= 14) (A), sperm morphology (■ n= 12, ■ n= 9) (B) and spermatogonia count (■ n= 10, ■ n= 9) (C) of RA and CS-exposed fathers. Immunofluorescence staining (40x) and quantification of spermatogonia (Ki67: red, DAPI: blue) in the testes (white arrows; 120x) of RA and CS-exposed fathers (D). Data are compared by Unpaired student's *t* test (A,C) or Two-way ANOVA with Tukey's multiple comparisons test (B). Data are represented as mean ± SD. Experiments were performed two (B) and three (A,C,D) times independently, giving a total animals per group as stated above. Each data point represents an individual animal. RA: room air, CS: cigarette smoke, def.: defect.

Supplementary Table 1. Regulated spermatozoal miRNAs following paternal preconceptional smoking.

miRNA	Mean expression	log2 fold change	lfcSE	p-value	padj
miR-204-5p	353.17	-0.76	0.2206	0.0005	0.0315
miR-96-5p	332.66	0.65	0.2504	0.0094	0.0875
miR-340-5p	1411.07	1.06	0.3078	0.0006	0.0315

Effects of CS exposure on the regulation of miRNAs compared to controls. Positive fold change refers to higher miRNA expression in RA-exposed controls compared to CS-exposed fathers. Negative fold change refers to lower miRNA expression in RA-exposed controls and higher miRNA levels in CS-exposed samples. Data were generated from one experiment, giving a total of 4 (RA) and 5 (CS) animals. RA: room air, CS: cigarette smoke, log2-fold change standard error (lfcSE), adjusted p-value (padj).

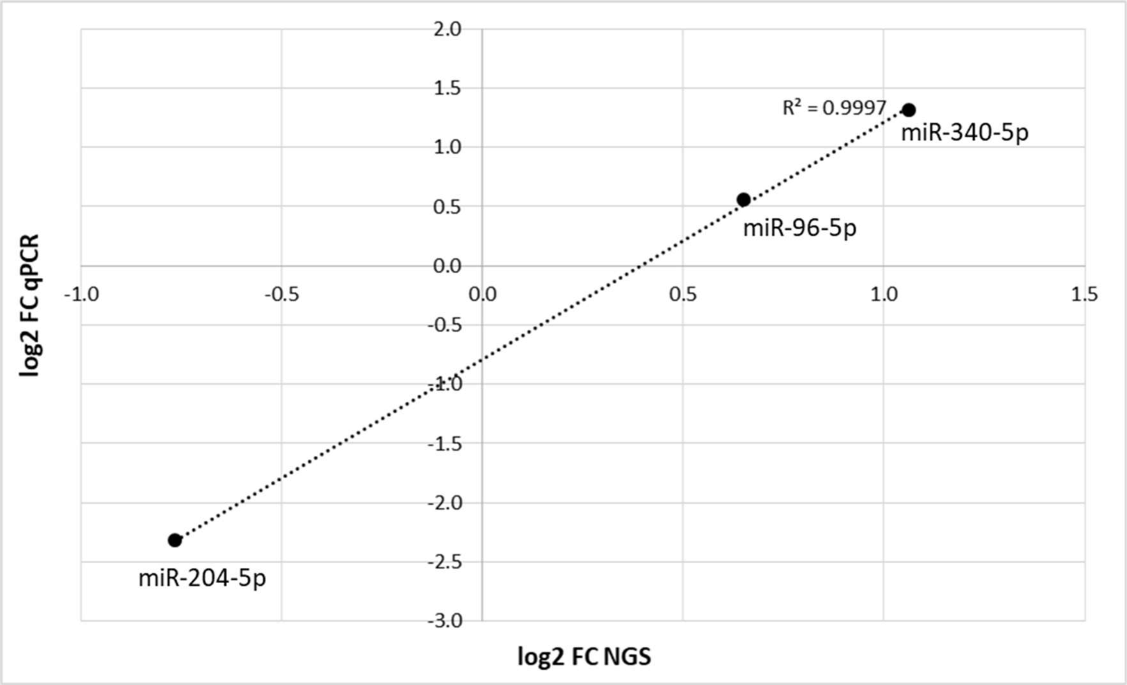

Supplementary Figure 4: Validation of regulated miRNAs identified by next-generation sequencing (NGS). Correlation of log2 fold changes (FC) of NGS data with RT-qPCR data of miRNA validation of miR-204-5p, miR-96-5p and miR-340-5p. Fold change (FC), miRNA (miR), next-generation sequencing (NGS), real-time quantitative polymerase-chain reaction (RT-qPCR). Data were generated from one experiment, giving a total of 4 (RA) and 5 (CS) animals per group.

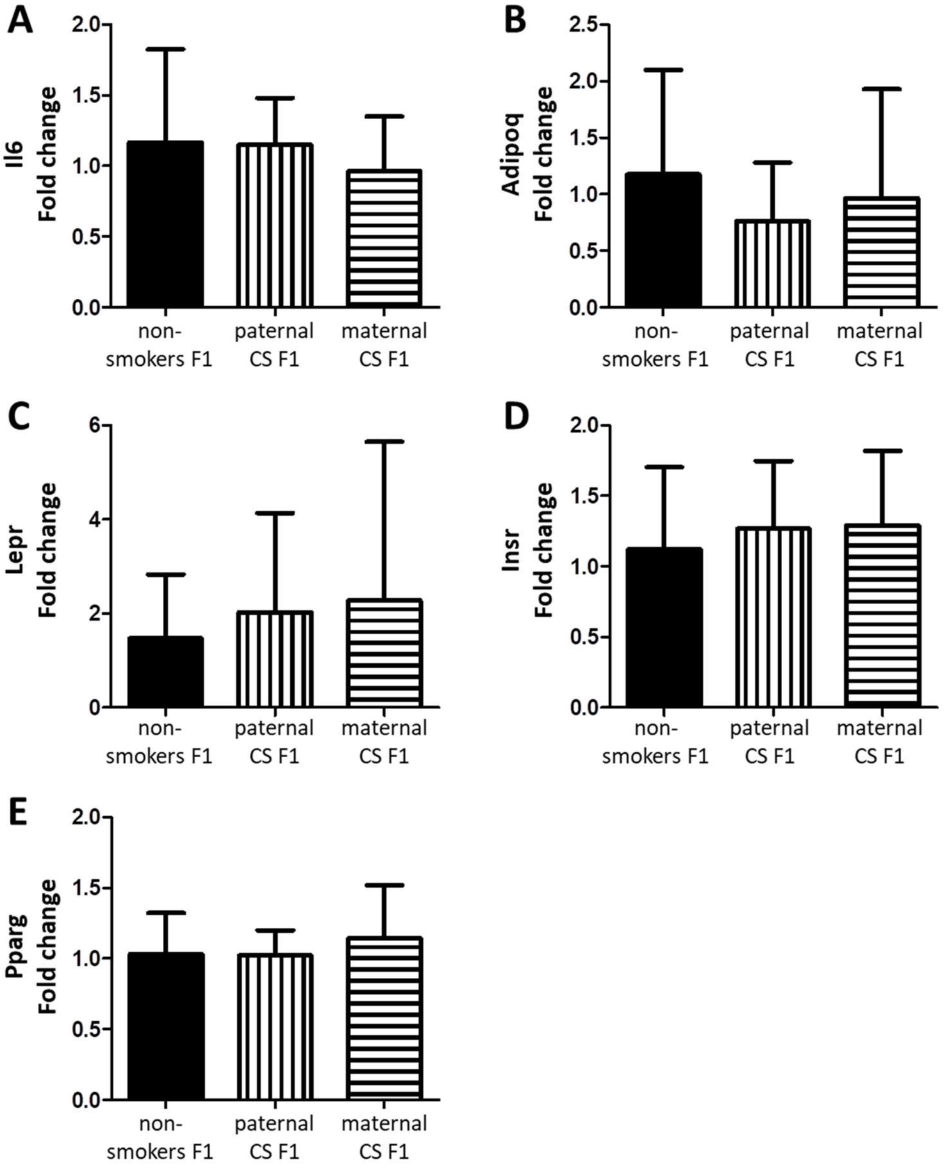

Supplementary Figure 5: Regulation of selected genes in the liver of offspring at PND21 after paternal and maternal preconceptional CS exposure. *Il6* (A), *Adipoq* (B), *Lepr* (C), *Insr* (D) and *Pparg* (E). Data were generated from one experiment giving a total of F1 n= 6 (non-smokers), F1 n= 8-10 (paternal CS), F1 n= 5-7 (maternal CS). Gene expression was normalized with HPRT and compared to non-smoker's offspring. Data are expressed as mean ± SD and compared by Mann-Whitney *U* test. F1: filial generation 1, CS: cigarette smoke. PND: postnatal day.

Supplementary methods

Animals and exposure

The study was approved by the government of the District of Schleswig-Holstein, Germany, [V244 – 77012/2016 (105-8/15)]. The inExpose exposure system (SCIREQ, Montreal, Canada) was employed for exposure of 3-week-old male and female C57BL/6J mice in a whole body chamber (maximum of 6 animals in the chamber) to room air (RA) or mainstream cigarette smoke (CS) (Research Cigarettes 3R4F, University of Kentucky, Lexington, Kentucky, USA) once per day for 1 h, 5 days/week. CS exposure began with 1 puff/min for 2 weeks and continued with 4 puffs/min for 4 weeks (**Supplementary Figure 1A**). Weights of offspring were recorded until postnatal day 21 (PND21) (Entris Precision Balance, Sartorius, Goettingen, Germany). RT-qPCR of offspring's liver was done using synthesized cDNA (LightCycler 480 II, Roche, Basel, Switzerland). Immunofluorescence staining of testes was performed with specific antibodies for Ki67 (Ki67 monoclonal antibody, clone: SolA15, eBioscience, San Diego, California, USA; polyclonal antibody Goat anti-Rat IgG (H+L) Cross-Absorbed Secondary Antibody, Alexa Fluor 546, Thermo Fisher Scientific, Waltham, Massachusetts, USA). Proliferation of spermatogonia were detected with a fluorescence microscope (Axio Observer.Z1, Zeiss; Axiocam MR, Zeiss, Oberkochen, Germany).

Spermatozoa isolation

Cauda epididymis and ductus deferens were dissected and processed in a sperm isolation medium (Dulbeccos's PBS with Ca, Mg, 5.6 mM D-glucose, 1 mM pyruvate and 5 mg/ml BSA, low endotoxin, pH 7.2). Spermatozoa were allowed to swim out for 10 min at 37°C, filtered (5 ml polyethylene round-bottom tubes with cell-strainer cap, VWR, Hannover, Germany) and counted with a hemocytometer. For morphology, 20x pictures were taken from Leja slides (SD-20-01-08-B, Nieuw-Vennep, the Netherlands) using an Olympus BX41 microscope (Waltham, Massachusetts, USA; DS-Ri1, Nikon, Tokyo, Japan).

Total RNA was isolated using miRNeasy Mini Kit (Qiagen, Hilden, The Netherlands) and RNA quality and integrity was analyzed with Agilent RNA 6000 Pico Kit (Bioanalyzer Agilent 2100, Agilent

Technologies, Santa Clara, California, USA), following manufacturer's instructions. High-quality RNA samples (260/280 ratio> 1.66, no degradation) were included in miRNA sequencing.

Next-Generation Sequencing and data analysis

100 ng total RNA were used for unbiased small RNA sequencing as described previously with minor modifications[(1)]. Briefly, sequencing libraries were constructed using the NEBNext Multiplex Small RNA Library Prep Kit (New England BioLabs, Ipswich, Massachusetts, USA) and amplified in 15 PCR cycles prior to size selection to extract fragments of 130–150 bp in length via a high-resolution agarose gel (4%). After careful library quality control via capillary electrophoresis, sequencing was performed on a HiSeq2500 Illumina (Illumina, New England BioLabs, San Diego, California, USA) using 50 cycles of single-end sequencing-by-synthesis [(2)].

Significant differentially expressed miRNAs were selected based on the criteria described in the publications Spornraft *et al.*[(3)] and Buschmann *et al.*[(1)].

Sequencing data were processed as described previously[(4)], FastQC (https://www.bioinformatics.babraham.ac.uk/projects/fastqc, version 0.10.1) was used to evaluate length distribution and sequencing quality prior to removing adaptor sequences via Btrim[(5)]. Furthermore, reads without detectable adaptor and short reads (< 16 nt), probably resulting from breakdown of longer RNAs, were removed. Additionally, reads that mapped to murine rRNA, tRNA, snRNA and snoRNA sequences downloaded from RNAcentral[(6)] were discarded. Next, remaining reads were aligned to murine miRNAs downloaded from miRBase (version 21)[(7)] using Bowtie's "best" alignment parameter and allowing one mismatch[(8)].

The Bioconductor Package DESeq2 (version 1.8.1)[(9)] was used to analyze differentially expressed miRNAs utilizing its built-in normalization strategy and false discovery correction (Benjamini–Hochberg method). To identify significantly regulated miRNAs, thresholds were set to a log2 fold change ≥|1,| an adjusted p-value of ≤0.05 and a mean expression of baseMean ≥50.

Mean Phred scores were 38.9 ± 0.7 for RA and 38.9 ± 0.6 for CS samples, reflecting excellent sequencing quality (Supplementary Table 2). Total libraries comprised 5.5E+06 ± 8.2E+05 reads for RA and 7.0E+06 ± 1.4E+06 reads for CS samples, out of which 1.4E+06 ± 1.6E+05 reads for RA and

1.5E+06 ± 3.6E+05 mapped to miRNAs (Supplementary Table 2). Less than 0.5% of reads in all samples carried no detectable adaptor, validating precise size selection during library preparation. 396 (RA) and 387 (CS) distinct miRNAs were detected with at least 10 mean per-group reads in RA and CS samples. After DGE analysis, 45 miRNAs were detected as significantly regulated, which were further used for the downstream analyses.

Supplementary Table 2. miRNA NGS: Mapping statistics.

	RA [mean]	RA [SD]	CS [mean]	CS [SD]
miRNA	1.4E+06	1.6E+05	1.5E+06	3.6E+05
tRNA	1.5E+06	1.9E+05	2.3E+06	5.3E+05
snoRNA	2.6E+04	1.8E+03	3.4E+04	6.5E+03
snRNA	1.1E+04	4.0E+03	9.9E+03	2.8E+03
rRNA	8.5E+05	2.4E+05	1.2E+06	1.5E+05
Unmapped	1.5E+06	5.2E+05	1.7E+06	3.6E+05
Short	2.2E+05	2.9E+04	2.2E+05	5.7E+04
No adaptor	2.0E+04	3.2E+03	2.2E+04	7.9E+03
Library size	5.5E+06	8.2E+05	7.0E+06	1.4E+06
No Adaptor [%]	0.4		0.3	

Quantitative real-time polymerase-chain reaction

miRNAs selected from NGS data were validated using TaqMan miRNA assays (Rack ID: P02998841, Applied Biosystems, Thermo Fisher Scientific, Waltham, Massachusetts, USA). 10 ng total RNA used for the NGS analyses of miRNAs were synthesized to 50 µl cDNA (TaqMan Advanced miRNA cDNA Synthesis Kit, Thermo Fisher Scientific, Waltham, Massachusetts, USA) using specific reverse transcription (RT) primers for the miRNAs (TaqMan MicroRNA Assays: miR-96-5p, lot number P190424-009 D05; miR-204-5p, lot number P190310-001 D06; miR-340-5p, lot number P190724-007 F02; U6, lot number P191009-014 D10). After optimization routines and using commercially acquired primers (TaqMan MicroRNA Assays), 2.5 µl of undiluted cDNA was used within a total volume of 10 µl for RT-qPCR (LightCycler TaqMan Master, LightCycler 480 II, Roche, Basel, Switzerland) following manufacturer's instructions. Ct values of miRNAs were normalized to those of U6 and relative miRNA expressions further calculated for samples from RA and CS groups with the ΔΔCt method.

Statistical analyses

Data were tested for normal distribution with D'Agostino-Person omnibus normality test. Normally distributed data were analyzed by Unpaired student's *t* test (t test) or more than two groups were tested with one-way analysis of variance (ANOVA, Tukey post-hoc test). Mann-Whitney *U* test (MWU) or Kruskal-Wallis test with Dunn's correction were applied to not normally distributed data. For two-factor comparison, two-way ANOVA was applied (Sidak's or Tukey' post-hoc test).

KEGG and GO analyses

MicroT-CDS algorithm was used to identify miRNA gene targets using the mirPath tool (v.3).[10] KEGG pathway and Gene Ontology analyses were run on targeted genes. Both "a priori" and "a posteriori" analyses have been undertaken. In "a priori" analysis, two gene lists were produced: a list implementing the union of all targeted genes and another list with the intersection of genes targeted by at least five miRNAs. KEGG pathway and ontology enrichment analyses were run on produced gene lists. On the other hand, "a posteriori" union analysis performs the enrichment analysis and calculates the significance levels (p-values) between each miRNA and every pathway. Subsequently, for each pathway, a merged p-value is calculated by combining the previously calculated significance levels, using Fisher's meta-analysis method.

Raw sequencing data are accessible at European Nucleotide Archive under the accession number PRJEB35967.

Linear mixed-effect model

Linear mixed-effect models are linear regression models which incorporate fixed effects (time-independent variables) and random effects (time-dependent variables) where both the intercept and the slope are allowed to vary for each subject. Such models are used for analysing hierarchical data with repeated measurements such as the mouse body weight data in this study. The longitudinal weight data are nested within mice which in turn are nested within litter. The body weight data for 21 days (dependent random variable) were set as level 1, the mice data as level 2 and litter data as level 3, thus accounting for variations in offspring's weight due to litter-specific factors. We generated a linear

mixed-effect model to our hierarchical mouse body weight data using mixed effect maximum likelihood regression method ('mixed' command of Stata 15 (Lakeway Drive, Texas, USA). Stata coding used for the multilevel modellings is available from the corresponding author on request.). This model allows the birth weight (intercept) and change in weight over time (slope) to vary at the individual offspring level.

Time-independent variables (fixed effects) were gender (male, female) and parental CS-exposure (none, paternal, maternal).

We fitted three models where the intercept was allowed to vary (random intercept model). The first model was an unconditional model with no measure of time or fixed-effect factors (gender, exposure). The second model was a random-slope model which incorporated a measure of time (Days). The third model incorporated the time factor and was adjusted for fixed-effects (mixed-effect model). The goodness of fit of each of the models was checked using the likelihood ratio test. The results presented are from the model with the best fit and the lowest intra class correlation (see below):

$Weight_{it} \sim days_{it} + exposure_i + sex_i$ || litter || mice: days

where i = 1, 2, …., 72 mice and t = 0, 1,…., 21 days

REFERENCES

1. Buschmann D, Kirchner B, Hermann S, Marte M, Wurmser C, Brandes F, et al. Evaluation of serum extracellular vesicle isolation methods for profiling miRNAs by next-generation sequencing. J Extracell Vesicles. 2018;7:1481321.
2. Mardis ER. Next-generation DNA sequencing methods. Annu Rev Genomics Hum Genet. 2008;9:387-402.
3. Spornraft M, Kirchner B, Haase B, Benes V, Pfaffl MW, Riedmaier I. Optimization of extraction of circulating RNAs from plasma--enabling small RNA sequencing. PLoS One. 2014;9:e107259.
4. Hermann S, Buschmann D, Kirchner B, Borrmann M, Brandes F, Kotschote S, et al. Transcriptomic profiling of cell-free and vesicular microRNAs from matched arterial and venous sera. J Extracell Vesicles. 2019;8:1670935.
5. Kong Y. Btrim: a fast, lightweight adapter and quality trimming program for next-generation sequencing technologies. Genomics. 2011;98:152-3.
6. Consortium RN, Petrov AI, Kay SJE, Gibson R, Kulesha E, Staines D, et al. RNAcentral: an international database of ncRNA sequences. Nucleic Acids Res. 2015;43:D123-9.
7. Kozomara A, Griffiths-Jones S. miRBase: annotating high confidence microRNAs using deep sequencing data. Nucleic Acids Res. 2014;42:D68-73.
8. Langmead B, Trapnell C, Pop M, Salzberg SL. Ultrafast and memory-efficient alignment of short DNA sequences to the human genome. Genome Biol. 2009;10:R25.
9. Love MI, Huber W, Anders S. Moderated estimation of fold change and dispersion for RNA-seq data with DESeq2. Genome Biol. 2014;15:550.
10. Vlachos IS, Zagganas K, Paraskevopoulou MD, Georgakilas G, Karagkouni D, Vergoulis T, et al. DIANA-miRPath v3.0: deciphering microRNA function with experimental support. Nucleic Acids Res. 2015;43:W460-6.
